# Digital Health Technologies for Long-term Self-management of Osteoporosis: Systematic Review and Meta-analysis

**DOI:** 10.2196/32557

**Published:** 2022-04-21

**Authors:** Ghada Alhussein, Leontios Hadjileontiadis

**Affiliations:** 1 Department of Biomedical Engineering Khalifa University of Science and Technology Abu Dhabi United Arab Emirates; 2 Healthcare Innovation Center Khalifa University of Science and Technology Abu Dhabi United Arab Emirates; 3 Department of Electrical and Computer Engineering Aristotle University of Thessaloniki Thessaloniki Greece

**Keywords:** mHealth, digital health, osteoporosis, self-management, systematic review, meta-analysis, chronic disease, bone health, self-management, nutrition, physical activity, risk assessment, mobile phone

## Abstract

**Background:**

Osteoporosis is the fourth most common chronic disease worldwide. The adoption of preventative measures and effective self-management interventions can help improve bone health. Mobile health (mHealth) technologies can play a key role in the care and self-management of patients with osteoporosis.

**Objective:**

This study presents a systematic review and meta-analysis of the currently available mHealth apps targeting osteoporosis self-management, aiming to determine the current status, gaps, and challenges that future research could address, as well as propose appropriate recommendations.

**Methods:**

A systematic review of all English articles was conducted, in addition to a survey of all apps available in iOS and Android app stores as of May 2021. A comprehensive literature search (2010 to May 2021) of PubMed, Scopus, EBSCO, Web of Science, and IEEE Xplore was conducted. Articles were included if they described apps dedicated to or useful for osteoporosis (targeting self-management, nutrition, physical activity, and risk assessment) delivered on smartphone devices for adults aged ≥18 years. Of the 32 articles, a random effects meta-analysis was performed on 13 (41%) studies of randomized controlled trials, whereas the 19 (59%) remaining studies were only included in the narrative synthesis as they did not provide enough data.

**Results:**

In total, 3906 unique articles were identified. Of these 3906 articles, 32 (0.81%) articles met the inclusion criteria and were reviewed in depth. The 32 studies comprised 14,235 participants, of whom, on average, 69.5% (n=9893) were female, with a mean age of 49.8 (SD 17.8) years. The app search identified 23 relevant apps for osteoporosis self-management. The meta-analysis revealed that mHealth-supported interventions resulted in a significant reduction in pain (Hedges *g* −1.09, 95% CI −1.68 to −0.45) and disability (Hedges *g* −0.77, 95% CI −1.59 to 0.05). The posttreatment effect of the digital intervention was significant for physical function (Hedges *g* 2.54, 95% CI −4.08 to 4.08) but nonsignificant for well-being (Hedges *g* 0.17, 95% CI −1.84 to 2.17), physical activity (Hedges *g* 0.09, 95% CI −0.59 to 0.50), anxiety (Hedges *g* −0.29, 95% CI −6.11 to 5.53), fatigue (Hedges *g* −0.34, 95% CI −5.84 to 5.16), calcium (Hedges *g* −0.05, 95% CI −0.59 to 0.50), vitamin D intake (Hedges *g* 0.10, 95% CI −4.05 to 4.26), and trabecular score (Hedges *g* 0.06, 95% CI −1.00 to 1.12).

**Conclusions:**

Osteoporosis apps have the potential to support and improve the management of the disease and its symptoms; they also appear to be valuable tools for patients and health professionals. However, most of the apps that are currently available lack clinically validated evidence of their efficacy and focus on a limited number of symptoms. A more holistic and personalized approach within a cocreation design ecosystem is needed.

**Trial Registration:**

PROSPERO 2021 CRD42021269399; https://tinyurl.com/2sw454a9

## Introduction

### Background

Osteoporosis, or porous bone, is a serious chronic disease in which the density of bones is silently and progressively reduced, resulting in a more porous and fragile structure [1]. This disease takes a huge personal and economic toll on the world [2]. The disabilities caused by osteoporosis outweigh those caused by cancer and many other chronic diseases. Both men and women can develop osteoporosis; however, women are more susceptible to this disease [3]. This silent killer is estimated to affect 200 million women worldwide—approximately one-tenth of women aged 60 years, one-fifth of women aged 70 years, two-fifths of women aged 80 years, and two-thirds of women aged 90 years [4]. By 2050, the worldwide incidence of hip fractures in both men and women is projected to increase significantly compared with the current number of cases [5]. Moreover, it is suggested that most individuals at high risk of osteoporosis are not properly diagnosed and are neither identified nor treated. It is also noted that >40% of patients with osteoporosis drop out from exercise therapies [6], and between 40% and 70% of patients adhere to drug therapies [7], which is not the case in patients with cancer or cardiovascular diseases. Therefore, it is important to identify and treat patients at risk of fracture, not only by prescribing effective medications but also by equipping them with the information they need to take appropriate behavior to prevent the consequences of the disease. This will substantially reduce the long-term burden of osteoporosis. Reducing the risk of the first fracture from 8% to 2% can reduce the 5-year fracture incidence from approximately 34% to 10% [2,5].

The current landscape of a rapidly aging population, accompanied by multiple chronic conditions, presents numerous challenges to optimally supporting the complex needs of this group. Therefore, it is essential to find better and affordable alternatives to hospital and institutional care that can support older adults in their homes rather than moving them to health care providers. The use of health-related mobile apps, or mobile health (mHealth), has emerged as an important and useful tool for improving health outcomes in chronic disease self-management [8]. Self-management is a very effective factor that can enhance overall health; it encompasses tasks, such as goal setting, active motivation, self-monitoring, decision-making, problem solving, planning for and engaging in specific behaviors, self-evaluation, stress management and emotional regulation, coping with lapses and setbacks, and assertive communication [9]. These mHealth apps allow for effective communication between patients and physicians, better clinical decision-making, and improved patient outcomes. Moreover, mHealth apps can support people to manage their own health, promote healthy living, and have access to the necessary information when and where they need it. They also have a groundbreaking impact on the pharmaceutical and health care industry because of their faster, better, and cheaper health management benefits [10,11].

The number of apps available on the planet exceeds 8 million, of which 60% are available on both Android and iOS app stores [12]. As of 2017, there are 325,000 mHealth apps with an annual download of >3.7 billion [13]. This increase in demand has resulted mainly from the growing penetration of smartphones and the emergence of advanced technologies in the health care sector. Moreover, the adoption of mHealth is likely to increase further, especially because of COVID-19 [14] and in remote areas that lack hospitals and clinics [15,16]. Despite the increasing number of mHealth apps, a limited number have been dedicated to patients with osteoporosis, although it is a major worldwide health challenge. In addition, a few studies have focused on long-term self-management of osteoporosis, which extends throughout the patient’s life. Even with the availability of cost-effective and well-tolerated treatments for osteoporosis, there is still no appropriate self-management of the disease to prevent fractures [17]. The individual responsibility for health and self-management of chronic diseases has been a concept with growing interest during the past decades [18], and mHealth can be useful for this purpose.

### Objective

The motivation behind this systematic review stems from the fact that, to the best of our knowledge, there is no other review so far that explores mHealth apps dedicated to osteoporosis self-management available in both the web-based app market and in the research field. The present systematic review and meta-analysis were undertaken to come up with the identification of the current status of osteoporosis-related mHealth solutions, reveal any lack of functionalities, identify challenges and barriers, and propose recommendations for more personalized and effective remote health care monitoring and interventions. In this way, efforts toward the development and testing of a holistic mobile app to support patients at risk of or with osteoporosis are better informed. Osteoporosis self-management apps with a holistic approach should comprise a wide variety of features, including nutrition, physical exercise, medication, and performance monitoring, in addition to involving a wide spectrum of stakeholders, from rheumatologists to other health care professionals, and requiring patients to be well-informed and to take an active role in their own car, while providing an incentive for physicians to trust, integrate, and implement mHealth apps into their medical practice.

## Methods

### Data Sources and Searches

For this systematic review, published sources were identified by searching PubMed, Scopus, Web of Science, IEEE Xplore, and EBSCO databases. A comprehensive combination of keywords was used to have the maximum possible coverage: *Osteoporosis* AND *Technology* OR *mHealth* OR *eHealth* OR *Remote Care* OR *Digital health technologies* OR *smartphone* OR *mobile phone* OR *Mobile applications* OR *app* or *Self-Management* OR *Disease management* OR *Bone health*. The titles and abstracts of all records were examined, whereas the full text was screened only for the potentially relevant studies for final inclusion; any duplicates were removed. Table S1 in Multimedia Appendix 1 shows the search terms and results yielded from the different databases in detail.

### Study Selections

The inclusion criteria were original studies or research papers, including people (both male and female) aged ≥18 years with no mental health conditions. The selected studies evaluated digital health technology, primarily designed to support targeted patient communication, education, diagnosis, real-time monitoring, and empowerment in the form of mobile phone apps supported by other audiovisual technologies. Moreover, we considered studies that use intelligent wireless sensors to capture any critical vital signs to support patients with osteoporosis in the long-term self-management of the disease. We also included studies that proposed a design or framework for mHealth apps targeting patients with osteoporosis. As no mHealth apps dedicated to osteoporosis self-management were found before 2010, only full-text studies published in peer-reviewed journals and in English from January 2010 to May 2021 were included.

Studies with participants who had mental disorders were excluded. In addition to studies that did not have full text available, we eliminated reviews, posters, letters, and expert opinion publications. We also did not consider studies with technological interventions not targeting or not useful for osteoporosis self-management, those that had no clear relationship with osteoporosis, those related to other musculoskeletal conditions, or those not useful for osteoporosis. Articles that did not use mobile apps were excluded, in addition to studies that examined social network platforms and services (such as Telegram, Skype, WhatsApp, or Facebook), emails, and the web. In the same context, we excluded studies that did not use any mobile app or use mobile technologies as an auxiliary tool, namely, by sending SMS text messages to engage patients in certain activities or behaviors.

### mHealth App Selection

We searched for *osteoporosis*, *bone health*, and *fracture* in different web-based app stores, including Google Play Store, Apple Store (iTunes), Amazon App store, Samsung Galaxy store, and GetJar. We found 72 apps, among which we selected only apps that were in English, targeted patients with osteoporosis, and focused on health, fitness, nutrition, and health categories. We excluded apps that were in the games and entertainment categories, apps that only recorded users’ data without any feedback, and apps that provided access to magazine conferences or journals. The remaining apps were categorized according to the main features they provide, such as educational content, prediction and assessment tools, and users’ tracking of osteoporosis-related pain and symptoms or both.

### Data Extraction and Quality Assessment

The following information was abstracted from each study: sample size, sample age range, app name, app purpose, app operating platform, study design, intervention period, and major outcome indices. Publication bias of randomized controlled trial (RCT) studies was evaluated using the Cochrane risk of bias (ROB; version 2.0) tool [19], whereas the bias of the nonrandomized comparative studies was assessed using the ROB In Nonrandomized Studies of Interventions tool [20]. The latter comprises 7 domains to assess bias because of confounding factors, selection of participants, classification of interventions, deviation from intended interventions, missing data, measurement of outcomes, and selection of reported results. The ROB adjudications are categorized with their corresponding color schemes as follows: low risk (green), moderate risk (yellow), serious risk (orange), critical risk (red), or no information (gray).

The selection, screening, data abstraction, and quality appraisals were performed by 2 reviewers (GA and LH). Any disagreements between the reviewers were resolved through discussion.

### Data Synthesis and Statistical Analysis (Meta-analysis)

Data from 41% (13/32) of studies were pooled in a statistical meta-analysis using meta-essential [21]. A random effects model was performed for 10 outcomes to compare before and after mHealth app use; that is, calcium intake (mg per day), vitamin D intake (µg per day), bone mineral density (BMD) levels, physical activity (hours per week), pain intensity, disability, physical functioning, well-being, fatigue, and anxiety. For each included outcome, the Hedges adjusted *g* [22] effect size was calculated and reported with 95% CIs. Heterogeneity was statistically assessed using Cochrane Q [23] and *I*^2^ tests, with high values (*I*^2^>50%) indicating high heterogeneity [24]. A 2-tailed *P*<.05 was considered significant in all the analyses. Statistical analyses were performed using random effects models. Moreover, statistical findings from the remaining 59% (19/32) of studies included in the review were narratively interpreted.

On the basis of the features provided by the apps, a scoring system was created for each app from the web-based market and those included from the research field. The selection of the scoring features stemmed from a combination of related theories. In particular, we followed the Technology Acceptance Model [25], which emphasizes the key factors that predict technology adoption by an individual based on the perceived usefulness and ease of use of the related technology, in our case, mHealth apps. Consequently, features that reflect the usefulness and ease of the proposed osteoporosis-related apps were considered. Moreover, features such as aesthetics and minimalistic design, recognition rather than recall, and error prevention were selected based on the 10 usability heuristics for user interface design of Nielsen [26,27], which were considered to be among the most frequent defects in mobile apps. Furthermore, many mHealth apps are used for consumption of a healthy diet, disease diagnosis, tracking physical activities, calculating calories, and monitoring sleep quality [28,29]. As these categories have already been accepted as interesting features in mHealth apps and are considered useful, we included them in the proposed analysis. Other features, such as notifications and reminders, create a sense of emotional bonding between users and the mHealth app, allowing them to keep self-managing their disease and, therefore, apply better monitoring of its symptoms and current health status [28]. Data sharing with designated individuals is another important feature that users consider important in any mHealth app [30]. In this way, a holistic perspective of the necessary features that could benefit the usefulness, easiness, user engagement, and plan adherence was followed. Apparently, as the selected features were spread across the different apps and were evaluated by different end users, no weighting process was applied. This allowed for an objective basis of scoring analysis across all studies toward the maximization of the integration and competence of the apps’ features. A feature weighting process would be useful if the focus of the analysis was placed on specific app functionalities or if an app could accommodate all features in an integrated way and be evaluated by end users, providing a rating of each feature’s significance.

**Figure 1:**
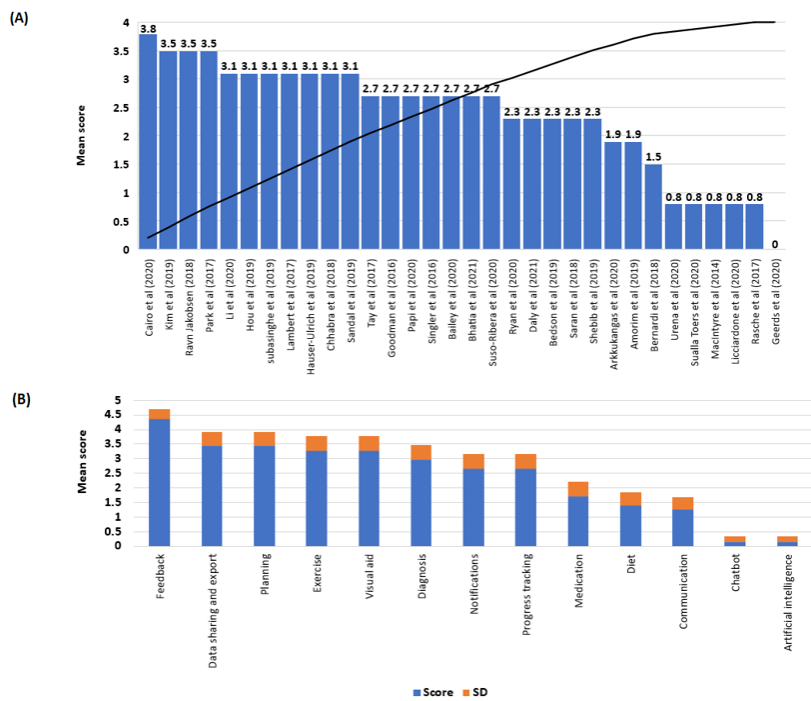
Scoring for research apps: (A) Mean score per app available in the literature, with a raw cutoff score of 2.7; apps above the threshold provide a more holistic self-management plan. (B) Selected features with their mean score representing how often they were present in the apps. Features with the highest scores were available in a larger number of apps; features with the lowest scores (ie, chatbot and artificial intelligence) were present in only 1 app [31-59].

**Figure 2:**
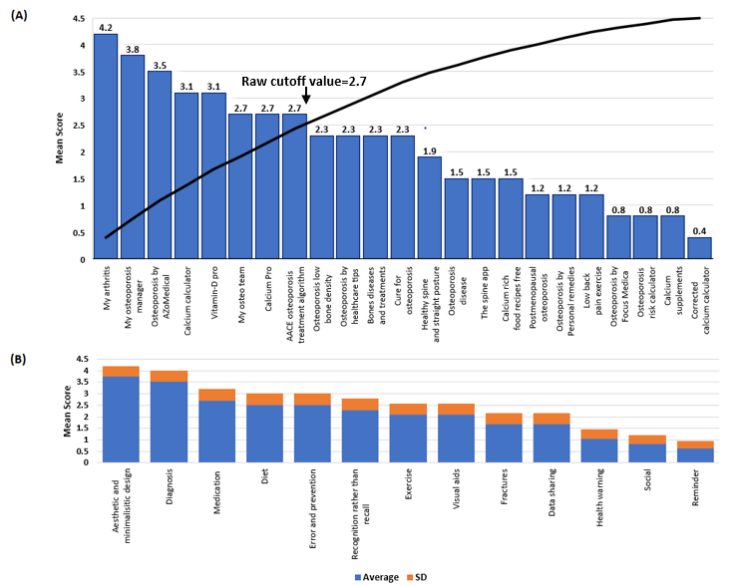
Web-based apps scores: (A) Mean score per app available in the web-based markets, with a raw cutoff score of 2.7; apps above the threshold provide a more holistic self-management plan. (B) Selected features with their mean score representing how often they were present in the apps. Features with the highest scores were available in more apps, whereas the features with the lowest scores were present in only 2 to 3 apps.

Table 1 provides an overview of the selected features and how they relate to self-management. For the web-based apps, 13 features were considered. These features were (1) diagnosis, (2) diet, (3) medication, (4) fractures, (5) error prevention (helping users recognize, diagnose, and recover from errors), (6) exercise, (7) visual aids, (8) data sharing, (9) social network, (10) reminders, (11) health warnings, (12) aesthetic and minimalistic design (avoid providing irrelevant or rarely required information), and (13) recognition rather than recall (remember user’s choices and visible and easily retrievable instructions of use). Similarly, 13 features were evaluated for the research apps. These were (1) diet, (2) exercise, (3) diagnosis, (4) medication, (5) data sharing or export, (6) planning, (7) notifications, (8) chatbot, (9) visual aids, (10) progress tracking, (11) feedback, (12) communication, and (13) artificial intelligence (AI). The presence of each feature added 1 point to the total accumulated score for each app. The accumulated score (out of 13) was converted to a score out of 5. Finally, these scores were ranked in decreasing order (holistic osteoporosis management apps to atomistic apps). The raw cutoff for the selection of the selected studies or app was determined based on the mean score versus studies cumulative plot (Figures 1 and 2).

**Table 1:** Self-management features for both research and web-based apps.

Self-management facet	Web-based market app feature	Research app features
Socialization	Networking capabilities Data sharing	Data sharing or export Communication
Scheduling	Reminders Medication plan Diet programs Exercises	Planning Medication plan Diet programs Exercises
Warnings	Fractures Health warnings	Notifications
User acceptability and usability	Visual aids Aesthetic and minimalistic designa Recognition rather than recalla Error prevention^a^	Visual aids
Personalization or adaptation to change	N/A^b^	Chatbot Artificial intelligence
Performance monitoring	N/A	Feedback Progress tracking
Self-care	Diagnosis	Diagnosis

^a^These features were selected based on the 10 usability heuristics for user interface design of Nielsen and Mack [26], which were considered to be among the most frequent defects in mobile apps. It was not possible to evaluate some usability features in the research apps as they were not publicly available in app stores.

^b^N/A: not applicable.

This systematic review was performed based on the recommendations of the PRISMA (Preferred Reporting Items for Systematic Reviews and Meta-Analyses) statement [60]. The PRISMA checklist is provided in Multimedia Appendix 1, Table S2. The methods of analysis and the inclusion criteria were specified in advance.

## Results

### Literature Search Results

**Figure 3:**
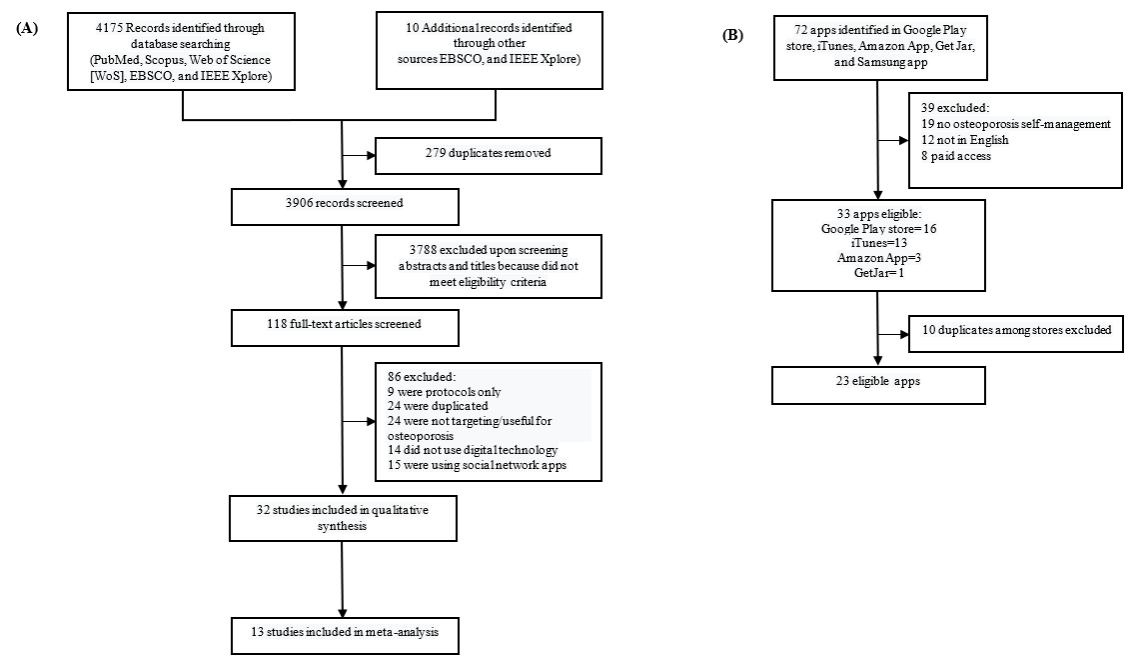
Flow diagrams for the selection of (A) studies and (B) apps.

The literature search yielded 4185 articles, of which 3906 (93.33%) were screened. After removing duplicates and excluding studies on the basis of their titles and abstracts, 3.02% (118/3906) full texts were assessed for eligibility. In the final stage, 74.6% (88/118) of full-text citations did not meet the inclusion criteria. After completely reviewing the corresponding full-text articles, of the 88 articles, the total number of accepted articles was reduced to 32 (36%), of which 13 (41%) were selected for the meta-analysis. A PRISMA flowchart [60] for the article selection and exclusion process is provided in Figure 3A. We conducted an in-depth review of each of the included articles to classify them according to their research findings and determine their current state of knowledge. The data extracted from the selected papers are shown in Table 2.

**Table 2:** Research app characteristics.

Author	App name	Sample size (age)	Experiment (participant sample size)	Platform^a^ (private or public)	App purpose (direct or indirect)	Intervention period	Major outcome indices
Daly et al [38]	PhysiApp-patient portal	20 (>65 years)	App (20)	Android (public)	Remotely delivers and monitors an individually tailored, home-based multicomponent exercise program (indirect^b^)	8 weeks	Feasibility, usability, physical activity enjoyment, changes in lower extremity function, and level of physical activity
Bhatia et al [44]	Manage My Pain	246 (mean age 57, SD 15 years)	App (111); no app (135)	Android and iOS (public)	Measures and monitors pain, function, and medication use (indirect)	92-183 days	Anxiety, depression, pain catastrophizing, satisfaction, daily opioid consumption, engagement
Cairo et al [59]	Vida app	127 (>18 years)	App (66); no app (61)	Android and iOS (public)	Improves wellness outcomes for survivors of breast cancer (indirect)	6 months	Physical activity, diary patterns, fatigue, and depression improvement
Hauser-Ulrich et al [53]	SELMA-Chatbot	102 (mean age 43.7 years)	App (59); no app (43)	Android and iOS	Promotes self-management of chronic pain (indirect)	12 weeks	Pain-related impairment, intention to change behavior, and pain intensity
Suso-Ribera et al [51]	Pain Monitor	87	App (43); no app (44)	N/A^c^ (private)	Improves existent medical treatments for patients with chronic musculoskeletal pain (indirect)	4 weeks	Pain severity and interference, fatigue, depressed mood, anxiety, and anger
Licciardone et al [36]	N/A	102 (mean age 51 years)	App (52); no app (50)	N/A	Self-management of health‐related quality of life (indirect)	3 months	Change in the SPADE^d^ cluster score, changes in low back pain intensity, and back‐related disability
Geerds et al [35]	N/A	24 (older adults >60 years)	App (24); no app (24)	N/A (private)	Monitors postoperative functional outcome after hip fracture (indirect)	12 and 18 weeks after surgery	Usability
Bailey et al [47]	Hinge Health app	10,264 (mean age 43.6 years)	App (10,264)	N/A (private)	Provides education, sensor-guided exercise therapy, and behavioral health support with one-on-one remote health coaching (indirect)	12 weeks	Pain measured by the Visual Analog Scale, engagement levels, program completion, program satisfaction, condition-specific pain measures, depression, anxiety, and work productivity
Ryan et al [48]	Striving app, Boning up	290 (40-60 years)	App (84); e-book (84); no app (84)	Android and iOS (private)	Provides information and feedback and monitors behavior change (direct^e^)	12 months	Bone mineral density and trabecular bone scores
Papi et al [32]	Nymbl	35 (≥55 years)	App (35)	N/A (private)	Trains balance in the older population (indirect)	3 weeks for all, with optional follow-up for 3 weeks	Physical activity level and adherence and IPAQ^f^ questionnaire
Sandal et al [45]	selfBack	51 (mean age 45.5, SD 15.0 years)	App (51)	N/A (private)	Improves self-management of low back pain (indirect)	6 weeks	Pain-related disability (RMDQ^g^) and multiple self-reported outcomes
Urena et al [33]	m-SFT	7 (53-61 years); the system usability was evaluated by 34 health experts (mean age 36.64 years)	App (7)	Android (private)	Easy-to-use tool for a health practitioner to record and assess the physical condition of older adults (indirect)	N/A	Usability questionnaire
Li et al [49]	Caspar Health App or Website	31 (≥60 years)	App (15); no app (16)	Android and iOS (public)	Postfracture telerehabilitation (direct)	3 weeks	Motor performance, functional performance, and fall efficacy; degree of independence in ADL^h^ performance
Kim et al [40]	Fracture Liaison Service	60 (>60 years)	App (60)	Android and iOS (public)	Fall prediction and monitoring (direct)	N/A	Usability
Amorim et al [37]	Fitbit (activity tracker) and IMPACT app	68 (mean age 58.4, SD 13.4 years)	App (34); no app (34)	Android and iOS (public)	Reduces care seeking, pain, and disability in patients with chronic low back pain after treatment discharge (indirect)	15 months	Care seeking, pain levels, and activity limitation
Subasinghe et al [57]	Tap4Bone: MyFitnessPal, Nike Training Club, and QuitBuddy	35 (mean age 23.1 years)	App (18); no app (17)	Android and iOS (public)	MyFitnessPal is a free calorie counter app that helps people track their diet and exercise; Nike Training Club is a free app comprising >100 full-body workouts; QuitBuddy is a smoking cessation internet-based app (indirect)	9 weeks	Feasibility and compliance
Arkkukangas et al [34]	OEP app	12 (70-83 years)	App (12)	N/A (private)	Fall prevention (indirect)	6 weeks	Questionnaire and behavior change
Shebib et al [50]	DCP with sensors	177 (mean age 43, SD 11 years)	App (113); no app (64)	N/A (private)	Aids self-management by engaging patients, and scales personalized therapy for patient-specific needs (indirect)	12 weeks	ODI^i^, Korff Pain, and Korff disability
Bedson et al [39]	Keele pain recorder	21 (>18 years)	App (21)	Android (public)	Records pain levels, interference, sleep disturbance, analgesic use, mood, and side effects (indirect)	28 days	Usability and acceptability
Hou et al [55]	eHealth	168 (18-64 years)	app (84); no app (84)	N/A (private)	Telerehabilitation and self-management interventions (indirect)	3, 6, and 12 months	Disease-specific questionnaire (ODI), Visual Analog Scale to record back pain, measures of mental health and life status, which included the EuroQol 5-Dimension health questionnaire
Saran et al [46]	N/A	927 (20-80 years)	App (927)	N/A (private)	Monitors physical activity (indirect)	1 week	Home physical activity
Chhabra et al [54]	Snapcare	93 mean) age 41.4, SD 14.2 years)	App (45); no app (48)	Android (private)	Monitors patient’s daily activity levels and symptomatic profile (indirect)	12 weeks	Pain and disability
Jakobsen et al [31]	My Osteoporosis Journey	18 (50-65 years)	App (18)	Android and iOS (private)	Provides information and usability questionnaires (direct)	12 weeks	Satisfaction with the app and risk calculation
Lambert et al [56]	PhysiotherapyExercises	80 (34-59 years)	App (40); no app (40)	N/A (private)	Home exercise programs (indirect)	4 weeks	Self-reported exercise adherence, The Patient-Specific Functional Scale, degree of disability, and patient satisfaction with health care service
Rasche et al [43]	Aachen fall prevention app	79 (>50 years)	App (79)	Android and iOS (private)	Self-assessment of older patients at risk for ground-level falls (indirect)	1 year	Objective fall risk and the self-assessed subjective fall risk
Park et al [52]	*Strong bone, Fit body*	82 (<25 years; women)	App (36); no app (38)	Android (private)	Provides feedback and records activity and nutrition (direct)	20 weeks	Bone mineral density, minerals, biochemical markers, food intake diary, knowledge, health belief, and self-efficacy
Tay et al [41]	Calci-app	40 (18-25 years)	App (40)	Android and iOS (private)	Usability questionnaires (direct)	5 days	Dietary calcium intake
Goodman et al [58]	VDC-app	109 (18-25 years)	App (59)	iOS (private)	Provides information and feedback and monitors behavior change (direct)	12 weeks	Vitamin D intake, knowledge, perceptions of vitamin D, blood concentrations of 25(OH)D3
Singler et al [42]	AOTrauma’s orthogeriatrics	920 (health professionals)	App (920)	Android and iOS (public)	Delivers the app to surgeons, trainees, and other health care professionals to measure use and evaluate the impact on patient care (direct)	Web-based one-time evaluation	Rating of app and usability

^a^App is available to the public in app stores, or app is not available to the public in app stores.

^b^The study has an indirect relation to osteoporosis.

^c^N/A: not applicable.

^d^SPADE: sleep disturbance, pain, anxiety, depression, and low energy or fatigue.

^e^The study or app has a direct relation to osteoporosis.

^f^IPAQ: International Physical Activity Questionnaire.

^g^RMDQ: Roland-Morris Disability Questionnaire.

^h^ADL: activities of daily living.

^i^ODI: Oswestry Disability Index.

### Characteristics of the Included Studies

All selected articles were published in journals over the preceding 8 years (2014-2021), with a notable increase in publications since 2017. The publications comprised feasibility studies [31-39], design and development articles [40-42,61-63], and case studies [43-47]. Among these studies, 41% (13/32) of articles were RCTs [36,37,48-58]. Although most of the articles have a direct relation to osteoporosis [31,40-42,49,52,57,61-63], some of the selected articles refer to apps that are useful and indirectly related to osteoporosis; that is, they are not specifically designed for osteoporosis yet can be potentially useful in managing the disease [32,33,35-37,39,45,51,53-55,58]. The included mHealth apps can be classified into different research themes: (1) monitoring apps (tracking patients’ daily nutrition, exercises, and symptoms) [34-36, 38, 40, 41, 44, 46-48, 50-55, 57-59, 64], (2) assessment apps (providing health professionals and patients various tests for assessing patients) [32,33], and (3) measurement apps (measuring certain parameters or variables related to osteoporosis) [43,61-63]. Among all the selected studies, only one of the studies conducted by Ravn Jakobsen et al [65] used participatory design involving all stakeholders, including researchers, women, physicians, health care professionals, and app designers, in the design process of the app named *My Osteoporosis Journey*. After the development stage, they also presented the testing of their collaboratively designed app with women newly diagnosed with asymptomatic osteoporosis [31].

### Characteristics of the Included Apps From Web-Based Market

As of May 2021, we found 33 relevant apps for osteoporosis. Most of the apps identified were found in Google Play (16/33, 48%) and Apple stores (13/33, 39%). Approximately 9% (3/33) of apps were available in the Amazon app store, 3% (1/33) in the GetJar app store, and none in the Galaxy app store.

After removing the overlapping apps across stores, 70% (23/33) of unique apps remained (Figure 3B). Among them, 56% (13/23) were developed to provide educational content on osteoporosis. The educational content covered the diagnosis of the disease, exercises, medications, and diet. It varied among animated videos, recorded videos, short articles, guided audio, expert advice, and graphs.

Table 3 presents all the identified apps in the web-based stores with their main characteristics, including name, operating system, description, users, and classification.

**Table 3:** Web-based app characteristics.

App name	Operating system	Description	Users	Classification
AACE osteoporosis treatment algorithm^a^	iOS	Provides evidence-based information about the diagnosis, evaluation, and treatment of postmenopausal osteoporosis for endocrinologists, physicians in general, regulatory bodies, health-related organizations, and interested laypersons	Health care professionals	Information and education
Calcium Pro^a^	Android and iOS	Provides information about calcium, parathyroid, osteoporosis, and vitamin D issues; inputs test results for calcium, parathyroid hormone, and vitamin D; analyzes and graphs tests making them easy to understand; tracking tools show calcium and vitamin D levels over time and provide feedback about bone density status; a risk assessment tool for conditions associated with high blood calcium	Patients	Monitoring, education, and assessment
Vitamin-D Pro^a^	iOS	Analyzes and graphs current vitamin D levels, calcium levels, calcium versus parathyroid hormone, bone density, and osteoporosis; teaches how to interpret data and graphs; gives personalized suggestions for next steps; suggests what new blood tests may be necessary; gives topics to discuss with the physician	Patients	Assessment, monitoring, and education tool
Osteoporosis Low Bone Density Weak Bones Diet Help^a^	Android	Provides information about the causes, symptoms, treatment, and the type of diet that one should eat to improve bone density	Patients	Information and education
Bones diseases and treatments^a^	Android	Information about all bone diseases	Patients	Information and education
My Arthritis^a^	Android	Keeps track of symptoms and flares; it can also track diet, exercise, pain, sleep, mood, stress; provides paid training courses with videos, guided audio, and expert advice; sets reminders for appointments and medication; access and share medical records from anywhere; learn about community news, current research, and other information	Patients	Monitoring, assessment, and management
Calcium Calculator^a^ (by BC Dairy)	Android	Tool to assess, compare, and plan to introduce enough calcium in daily food	Patients	Monitoring, assessment, and education
Osteoporosis^a^ (by AZoMedical)	iOS	Provides regularly updated information and news on osteoporosis	Professionals and patients	News
My Osteoporosis Manager	iOS	Capture detailed information regarding user’s health in a digital journal; manage medications and treatments; track osteo-specific symptoms and side effects feedback as easy-to-understand charts that record test results and medication adherence; access patient education materials; share information with a health care provider	Patients	Monitoring, assessment, and management
Osteoporosis (by Focus Media)	Android	Animated videos for learning about osteoporosis disease	Patients	Information and education
Osteoporosis disease	Android	Information about causes, symptoms, treatment, and the type of diet that one should eat to improve bone density	Patients	Information and education
Osteoporosis (by health care tips)	Android	Information and education	Patients	Information and education
Postmenopausal Osteoporosis	Android	Helps in understanding the disease condition through animated videos; it gives an insight into the structure and formation of bones, changes with age, and hormonal levels, particularly during menopause; it also provides information on the onset of osteoporosis, measurement of bone density, treatment, and self-help guidelines	Patients	Information and education
Osteoporosis (by personal remedies)	Android	Comprehensive and actionable nutrition guidelines for how to deal with osteoporosis; recipes, food suggestions, alternative therapies, and remedies	Patients	Information and education
Calcium Supplements	Android	Information about calcium supplements, including who should take them, their health benefits, and potential risks	Patients	Information and education
Osteoporosis AR	Android	Demonstrates a different fictional patient profile using the augmented reality technique that illustrates patient insights, symptoms they are experiencing, and how these agonizing symptoms affect patient’s quality of life	Patients	Information and education
Cure for Osteoporosis	Android	Information about raloxifene	Patients	Information
Osteoporosis Risk Calculator	Android	A risk check that calculates whether the user is at risk of fracture or osteoporosis	Patients	Measurement and assessment tool
Hip Fracture Risk Calculator	iOS	Calculates whether the user is at risk of fracture or osteoporosis based on patient demographics	Patients	Measurement and assessment tool
Calcium Calculator	iOS	Calculate calcium intake daily	Patients	Measurement tool
My Osteo-Team	Android and iOS	A social network and support group for those living with osteoporosis; users can acquire practical tips to manage their life with osteoporosis and insights about treatment or therapies	Patients	Social network
Low back pain exercise	Android	Exercises to reduce low back pain	Patients	Information and education
The spine app	Android	Information about back pain	Patients	Information and education
Fracture	Android	Information about fracture prevention	Patients	Information and education

^a^Ranked according to their rating rates, with the highest-ranking rates on the top, and vice versa. The other apps did not have any ratings or reviews. The ranking rate did not reflect the number of times the app was downloaded, and there was no direct relationship between the number of times an app was downloaded and its rating.

### Mobile Apps Ranking Based on Features

The results of app ranking are presented in Figures 1 and 2. The apps that scored the highest score (equal to or above the raw cutoff, 2.7), both in research and in the web-based market, provided more features, thus, reflecting a more holistic management of osteoporosis and its symptoms [31,40,52,59]. Apps that scored lower had fewer features or were designed for a single purpose, such as measuring spine curvature [63] or BMD [61] (Figure 1A). Approximately 87% (28/32) of the apps provided feedback to users, and 69% (22/32) allowed users to share or export their data and to have an individualized plan based on their individual needs and health goals. Only 3% (1/32) of apps provided a chatbot [53], and 3% (1/32) used AI [45] (Figure 1B).

Similarly, apps in the web-based markets that attained large scores, such as *My Arthritis*, offered more features to assist patients in the management of the disease (Figure 2A). Approximately 75% (17/23) of the web-based apps had good aesthetic and minimalistic designs (simpler designs with the content being the focal points); 70% (16/23) of these apps were designed for diagnosis purposes (Figure 2B).

### ROB and Methodological Quality

**Figure 4:**
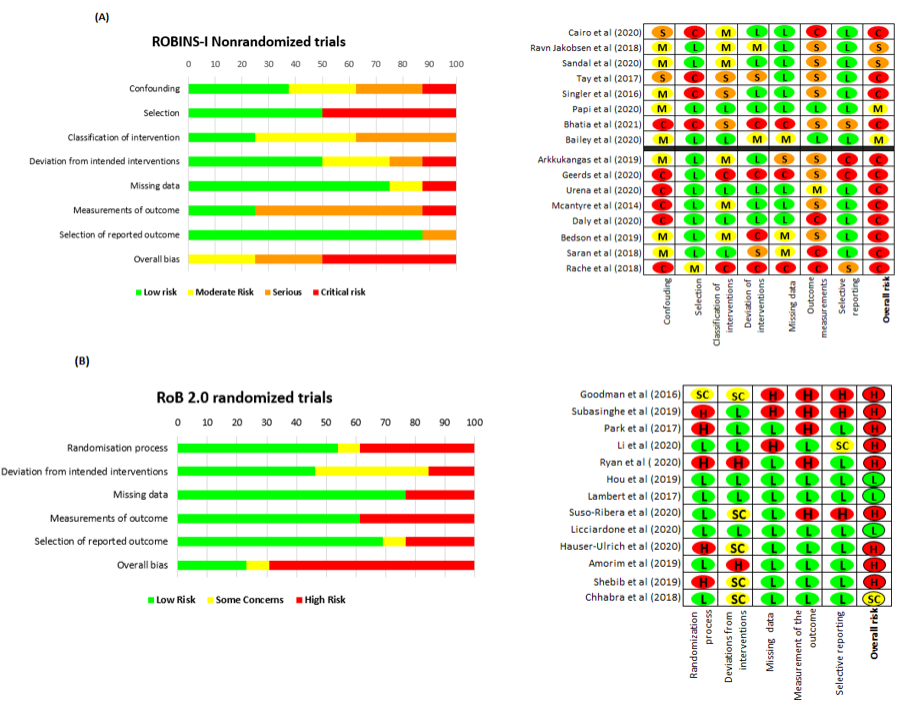
(A) Risk of bias (ROB) assessment for randomized (ROB 2.0) and (B) nonrandomized (ROBIN-I) trials. The studies above the horizontal black line are above the app's cutoff score (2.7) and vice versa [32,33,35-39,41-65]. ROBINS-I: ROB in Nonrandomized Studies of Interventions.

For the ROB In Nonrandomized Studies of Interventions assessment of nonrandomized clinical trials, 75% (12/16) of studies were at critical ROB, 13% (2/16) at serious risk, and 6% (1/16) at moderate ROB. Among the 13 RCTs assessed using the Cochrane ROB (version 2.0) tool [19], 3 (23%) studies showed a low ROB, and 1 (8%) study exhibited some concerns about the ROB. Approximately 69% (9/13) of RCTs showed a high ROB. Figure 4 summarizes the results of the bias and methodological quality assessments for all studies.

### Comparison Between Various Outcomes Before and After App Use

#### BMD T Score

**Figure 5:**
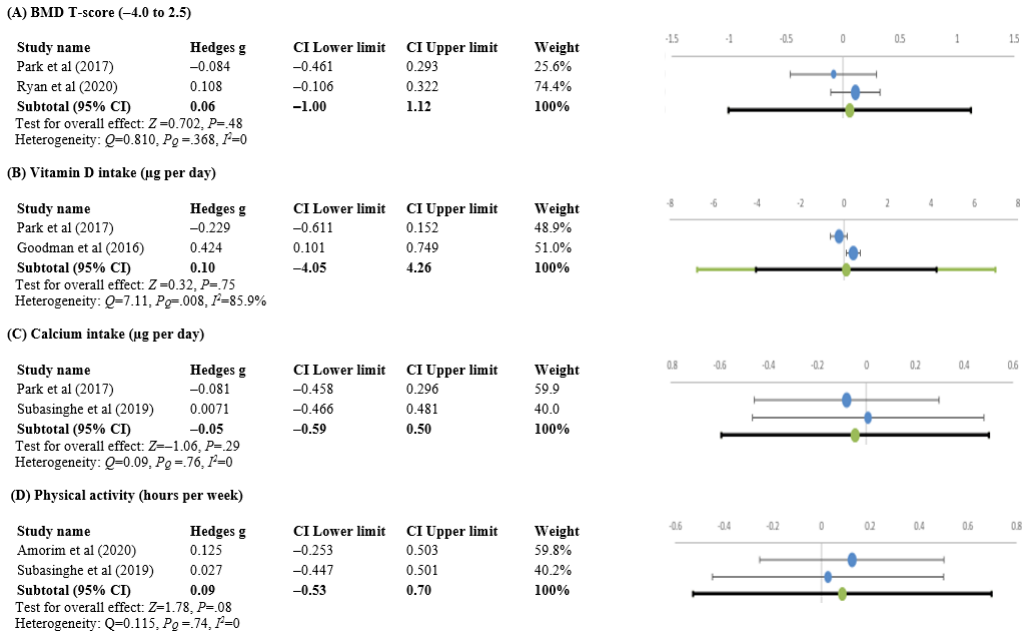
Forest plots of Hedges g effect size (95% CI) from individual studies before and after using the app showing changes in (A) bone mineral density (BMD) T score, (B) vitamin D intake (µg per day), (C) calcium intake (µg per day), and (D) physical activity (hours per week) [37,38,52,54-63].

Approximately 6% (2/32) of studies measured BMD T score at baseline and after 20 weeks [52] and 12 months [48] of using the apps. After initiation, a slight decrease in the mean BMD T score was observed in one of the studies (Hedges *g* –0.084, 95% CI –0.461 to 0.293) [52], and a slight increase was reported in another study (Hedges *g* 0.108, 95% CI –0.106 to 0.322) [48]. The overall change in mean T score was not significant (Hedges *g* 0.06, 95% CI –1.00 to 1.12; *Z*=0.702; *P*=.48), with no heterogeneity (*Q*=0.810; p_Q_=0.368; *I*^2^=0; Figure 5A).

#### Intake of Vitamin D (µg per Day) and Calcium (mg per Day)

Approximately 6% (2/32) of studies compared the average (µg per day) Vitamin D intake before and after app intervention [52,58]. There was a decrease in intake in one of the studies (Hedges *g* –0.229, 95% CI –0.611 to 0.152) [52], and a moderate increase in intake was observed in another (Hedges *g* 0.424, 95% CI 0.101 to 0.749) [58]. The overall change in intake was not significant (Hedges *g* 0.1, 95% CI –4.05 to 4.26; *Z*=0.32; *P*=.75), with heterogeneity among the studies (*Q*=7.11; p_Q_=0.008; *I*^2^=85.9%; Figure 5B).

Approximately 6% (2/32) of studies measured the differences in the average mg per day of calcium intake [52,57]. The daily intake of calcium did not differ significantly before and after app use (*Z*=−1.06; *P*=.29; Hedges *g* −0.05, 95% CI −0.59 to 0.50), with no heterogeneity (*Q*=0.09; p_Q_=0.762; *I^2^*=0; Figure 5C).

#### Physical Activity (Hours per Week)

Approximately 6% (2/32) of studies measured the average number of hours per week of physical activities before and after 15 months [37] or 9 weeks [57] of using the apps. After initiation of the intervention, there was no significant difference observed (*Z*=1.78; *P*=.08; Hedges *g* 0.09, 95% CI −0.53 to 0.70). There was no heterogeneity between the 2 studies (*Q*=0.115; p_Q_=0.735; *I*^2^=0; Figure 5D).

#### Physical Function

**Figure 6:**
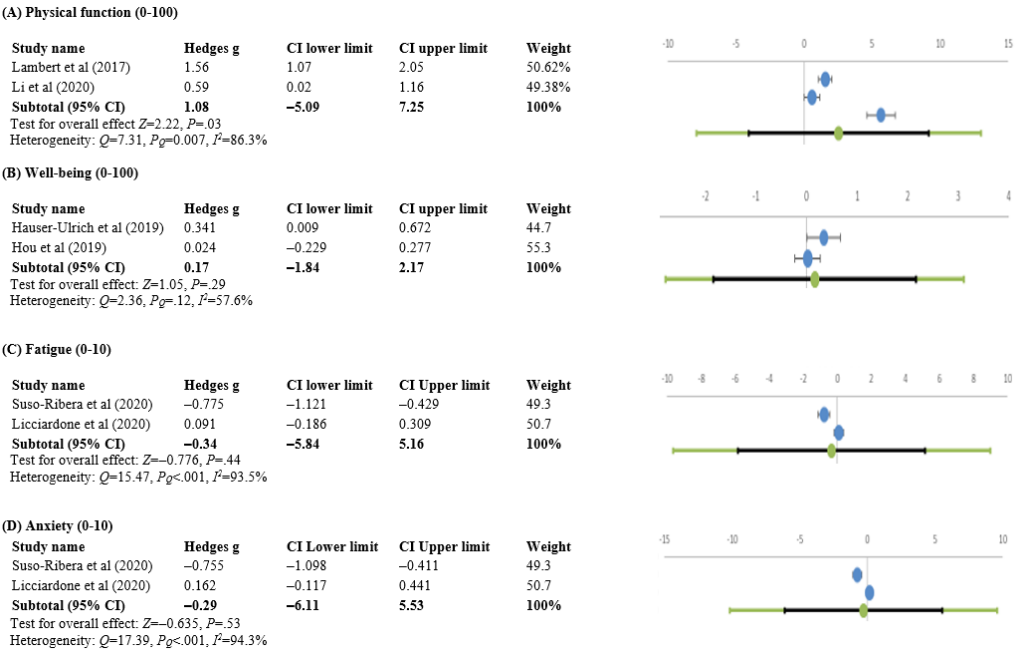
Forest plots of Hedges g effect sizes (95% CI) from individual studies before and after using the app showing changes in (A) physical function, (B) well-being, (C) fatigue, and (D) anxiety [37,38,53-55,57-60].

Approximately 6% (2/32) of studies evaluated physical function before and after 4 weeks [56] or 3 weeks [49] of using apps. There was a significant change in physical functioning at the end of the app interventions (Hedges *g* 1.08, 95% CI −5.09 to 7.25; *Z*=2.22; *P*=.03) with heterogeneity (*Q*=7.31; p_Q_=0.007; *I*^2^=86.3%; Figure 6A).

#### Well-being

Approximately 6% (2/32) of studies observed changes in well-being from baseline after 12 weeks [53] and 12 months [55] of using the apps. The improvement in well-being was nonsignificant (Hedges *g* 0.17, 95% CI –0.84 to 2.17; *Z*=1.05; *P*=.29), with no heterogeneity (*Q*=2.36; p_Q_=0.125; *I*^2^=57.6%; Figure 6B).

#### Anxiety and Fatigue

Approximately 6% (2/32) of studies measured changes in anxiety and fatigue at baseline and after 3 months [36] or 4 weeks [51] of intervention. The measured change was not significant for either anxiety (Hedges *g* –0.29, 95% CI –6.11 to 5.53; *Z*=–0.635; *P*=.53), with heterogeneity (*Q*=17.39; p_Q_=0; *I^2^*=94.3%), or fatigue (Hedges *g* −0.34, 95% CI −5.84 to 5.16), with heterogeneity (*Q*=15.47; p_Q_=0; *I*^2^=93.5%; Figures 6C and 6D).

#### Pain Intensity

**Figure 7:**
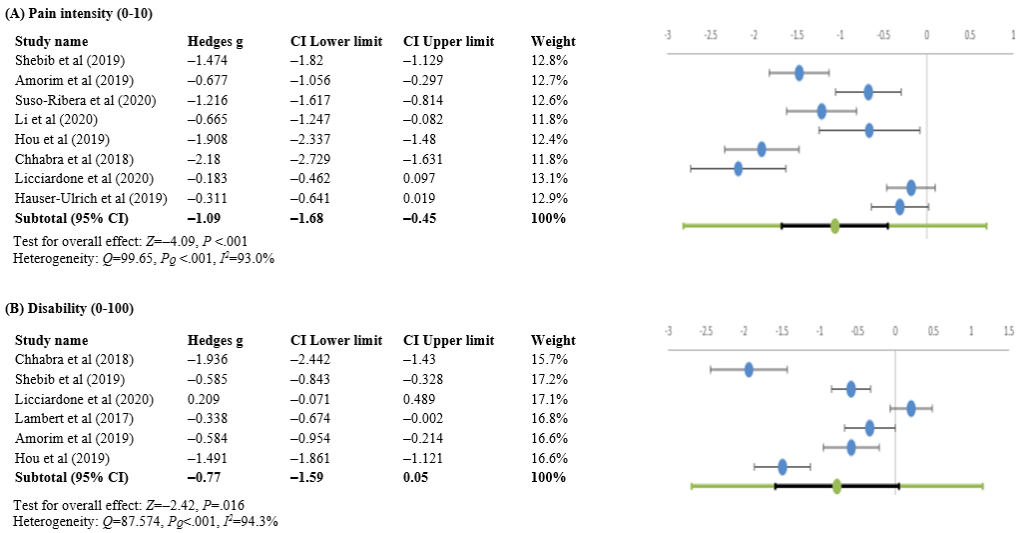
Forest plots of Hedges g effect sizes (95% CI) from individual studies before and after using the app showing changes in (A) pain intensity and (B) disability [37,53,55,57,59,60].

Approximately 25% (8/32) of studies recorded pain intensity before and after initiation of the interventions [36,37,49-51,53-55]. Overall, there was a significant decrease in pain across all studies (Hedges *g* –1.09, 95% CI –1.68 to –0.45; *Z*=−4.09; *P*<.001), with heterogeneity (*Q*=99.65; p_Q_=0; *I*^2^=93%; Figure 7A). A sensitivity analysis was performed to determine whether individual studies had a significant impact on the overall result. No significant differences (*P*=.81) were observed when excluding individual studies from the analysis (Multimedia Appendix 1, Table S3).

#### Disability

Approximately 19% (6/32) of studies evaluated disability [36,37,50,54-56]. The pooled estimate using the random effects model revealed significantly lower levels of disability (Hedges *g* –0.77, 95% CI –1.59 to 0.05; *Z*=−2.42; *P*=.02), with heterogeneity (*Q*=87.574; p_Q_=0; *I*^2^=94.3%; Figure 7B). The sensitivity analysis did not reveal any significant differences (*P*=.73); Multimedia Appendix 1, Table S4).

## Discussion

### Principal Findings

The focus of this review was placed on a systematic examination of the available literature on mHealth technologies and apps that can support the self-management of osteoporosis and decision-making for young and older adults. Although some of these apps showed promising results for the use of mHealth technologies in osteoporosis management, there is a lack of evidence in the research to prove the effectiveness of these apps, as validation studies have not been run on all the included apps.

Most (39/52, 75% apps) of the analyzed mHealth apps did not conduct premarket prospective multicenter RCTs. This might be because of the elevated cost of the trials and the long time required to recruit patients [66,67]. In addition, some apps did not publish evidence of their usability and acceptability among users [61,62].

From the scoring system created in Figures 1 and 2, it was possible to observe gaps in the provided features. For instance, only the apps available in the research fields provided feedback to the user, whereas this was not observed in the apps from web-based app stores. This raises an important issue regarding patient accessibility to their data and the overall functionality of these apps.

Our meta-analysis showed that by using the apps, pain scores were significantly reduced in 25% (8/32) of studies [36,37,50,51,53-55,64]. This finding was confirmed by 6% (2/32) of other studies, which found that apps can be beneficial for chronic pain management, especially for patients in an outpatient clinic setting [68,69]. The meta-analysis also showed reduced levels of disability, which is consistent with the findings of Briggs et al [70], who reported reduced disability in patients with osteoarthritis who used digital self-management interventions. In addition, we found that physical function significantly improved after using the apps [56,64].

According to our results, app use had no impact on the physical activity of app users. The meta-analysis also revealed that digital health interventions had no significant impact on the daily intake of calcium and vitamin D or on the BMD trabecular score. It is important to note that patients’ adherence to and compliance with the use of mHealth apps are pivotal in ensuring improved health outcomes and successful intervention programs. Some studies reported a high dropout rate in patients who found the intervention boring, time consuming [41,58], or infeasible for daily practice [35]. Another study pointed out that patient attrition led to nonsignificant results at the end of the study [41]. Therefore, any study should ensure to have a comprehensive retention plan for both experimental and control groups.

The data yielded by the meta-analysis demonstrated that using the app had no significant impact on well-being, anxiety, and fatigue scores. This might be explained by the fact that patients self-reported these outcomes in all the evaluated studies without any validation [37,39,44,47,50,54,71], and in many cases, they tended to exaggerate their symptoms in an attempt to prolong the intervention period [50]. To avoid problems arising from self-reporting outcomes, emotionally aware AI techniques could be applied to determine the behavior and emotional state of the user by interpreting their facial expressions while interacting with the app [72] or through emotionally aware chatbots [73].

A review of apps related to osteoporosis in the web-based marketplace resulted in 23 apps. Most of these apps provided information and education, such as disease definition, common symptoms, and suggested exercises to strengthen the bones and enhance physical activity or instructions on healthy nutrition. In addition, none of these available apps addressed the management of the disease after fracture, although fracture is the main complication of osteoporosis. Although osteoporosis is widespread in society, especially among adults, our findings revealed that the number of people who downloaded these osteoporosis-related apps is very limited, as it can barely reach 1000 downloads. Some of these apps did not report any downloads at all. This also indicates that patients or clinicians are hesitant toward the adoption of these new technologies. Unfortunately, these results revealed the poor contribution of research and development toward the field of mHealth apps designed for osteoporosis management and the untrustworthy content that does not have any strong reference [74].

Information privacy is an important issue in mHealth apps because of the sensitive nature of information gathered from users [75]. All identified apps in the web-based app stores were free, with pop-up advertisements every once in a while. Apparently, this creates a distraction for the user and sets some doubts about the way the collected data are used (eg, to create targeted advertisements according to the user’s profile). Apparently, this needs to be consented to by the user, following data privacy and security protocols, such as the General Data Protection Regulation [76].

Our findings show that only one of the identified studies [31] used a participatory design process to develop their app. Cooperative or participatory design involves stakeholders, designers, researchers, and end users in the early stages of the design process to ensure that the developed app meets the proper needs of its intended end users [77]. This entails that all stakeholders have equal input in the interaction design, which will nurture a more creative development atmosphere. Moreover, this cocreation session gives stakeholders a sense of ownership of the ideas that allow them to comprehend the thinking behind design decisions and improve their satisfaction levels. The involvement of health professionals in the design will also prevent any safety risks arising from inaccurate or unreliable digital tools [78].

### Overcoming Challenges in Osteoporosis mHealth Apps

#### Overview

This review shows that mHealth apps that use self-management support principles in primary care have the potential to have a positive effect on the management of chronic diseases. However, there is reluctance in the adoption of these digital technologies in health care. The main obstacles delaying the integration of these technological tools in osteoporosis care could be summarized as (1) weak or no involvement of health care professionals in the design process, (2) reluctance of clinicians who believe that mHealth apps might replace them, (3) lack of reliable tools and strict regulations, (4) privacy and security concerns, (5) data availability and visualization, (6) inconsistent data collection standards, (7) difficulties in acquiring and analyzing data, and (8) low retention rates of participants (Table 4).

**Table 4:** DRsa and CRsb for overcoming the identified limitations or barriers in digital health technologies for osteoporosis.

Identified limitation or barrier and related aspect			Recommendation
**App designers and developers without supporting information from clinicians, resulting in a very technologically focused and problem-oriented approach in the design of mHealth apps**			
	Design perspective	DR1: involve all the stakeholders in all the stages of user requirements, design, and development using a participatory design approach (cocreation)	
	Clinical perspective	CR1: active participation in the design, development, and testing stages	
**Clinicians’ reluctance in adopting mHealth** ^c^ **apps as they envisage that they will replace them**			
	Clinical perspective	CR1: adopt mHealth technologies in daily practices and in clinical care (measurement, assessment, and recording data) CR2: recommend trustworthy apps to their patients CR3: use mHealth apps to effectively communicate with patients and other health care professionals through the integration of wearables and IoTd	
**Lack of trustworthy and available smart tools and strict regulations on mHealth tools**			
	Design perspective	DR1: use adaptive learning algorithms (eg, AIe and machine or deep learning) in the app to make more personalized recommendations and treatments DR2: incorporate clinically validated monitoring, measurement, and assessment tools in the designed app	
	Clinical perspective	CR1: evaluate mHealth measurement and assessment tools by concerned clinical experts before disseminating them to public	
**Underestimation of the security risk and the elevated cost of implementing strong data security and privacy rules**			
	Design perspective	DR1: implement stringent security regulations (eg, GDPRf [79]) to protect users’ information from any data penetration (security and privacy by design)	
**Available data are provider oriented rather than patient accessible; limited existing guidelines on how to optimize user interfaces for patients, providers, or both**			
	Design perspective	DR1: allow patients to access their data (GDPR enforcement in design) DR2: generate feedback and plans (for diet and exercises) based on the gathered data to keep patients engaged and motivated	
**Inconsistent data collection standards, complexity of data, and lack of quality assurance processes (data cannot be verified)**			
	Design perspective	DR1: use passive and active gathering of data (medication, symptoms, nutrition management, and physical exercising), in addition to the data gathered from any wearables or IoT sensors	
	Clinical perspective	CR1: combine conventional clinical assessment with the app assessment	
**Difficulties in acquiring, analyzing, and applying structured and unstructured data to treat or manage diseases**			
	Design perspective	DR1: apply AI-based techniques that help with the prediction, diagnosis, and treatment or management of diseases	
**Low retention rates of participants**			
	Design perspective	DR1: provide valuable feedback to the user DR2: use simple and straightforward interfaces DR3: continuously update users’ data DR4: offer financial incentives for healthy habit changes	

^a^DR: design-related recommendation.

^b^CR: clinical recommendation.

^c^mHealth: mobile health.

^d^IoT: Internet of Things.

^e^AI: artificial intelligence.

^f^GDPR: General Data Protection Regulation.

To overcome these obstacles, we propose design-related and clinical recommendations for mHealth apps to support patients at risk of or diagnosed with osteoporosis in self-management and involve them in decision-making regarding treatment and intervention options with clinicians. These guidelines are not only limited to apps targeting osteoporosis self-management but can also be applied to any chronic disease self-management app.

#### Design-Related Recommendations

##### Co-design

Before creating an app, we should emphasize the role of end users, including patients and health professionals, in the development process. Users should be involved at various points and levels in the design process to improve their understanding of their needs, requirements, interactions, and appreciations before, during, and after developing the app. This co-design will ensure that the developed app meets end user purposes, leading to more effective results [80].

##### Integration of AI and Machine Learning in Data Acquisition and in Decision-making

An enhanced and intelligent version of the mHealth app can perform long-term management of osteoporosis through internet-based coaching using AI and big data analysis. In addition to health care professionals, AI can play an important role in the decision-making process and in the entire self-management process of osteoporosis. Conventional systems used for processing health data are less accurate and lack convergence compared with AI-supported systems [81]. Machine learning methods, more specifically adaptive learning algorithms, integrated into mHealth apps will make them tailored to an individual’s behavior and characteristics, thus improving the effectiveness of the intervention [82]. Such smart mHealth apps could unobtrusively acquire and effectively analyze sensorial and behavioral cloud-archived big data from adults’ interactions with smart devices (smartphones, smartwatches, and Internet of Things) in their daily living environment [83].

Owing to the significant advances and progress in AI in the past few years, chatbots have been gaining momentum in the eHealth world. Therefore, we believe that a bot framework can be incorporated with virtual reality technology and low-cost Internet of Things to create a user engagement schema for long-term monitoring of osteoporosis, where the patient will be active and maintain an improved quality of life.

##### Envisioning of a Smart Tool With User-Centered Orientation in Osteoporosis Management

An innovative technological tool (mHealth app) should offer an integrated platform for informed healthy living indoors or outdoors to assist patients with osteoporosis (or at risk) in different aspects of life, including physical activity, nutrition, medication intake, fall prevention, emotional wellness, and socialization. The design of such tools could include monitoring, combining both passive (via the interaction with smartphone or smartwatch or wearables) and active gathering of data (eg, about medication, nutrition management, and physical exercising). Then, on the basis of the gathered data, AI-driven data analysis processes could be involved in providing personalized feedback to the patient and informing the related physician, guiding personalized recommendations and interventions for osteoporosis risk assessment [84]. In this way, the patients will be kept aware of their progress in osteoporosis self-management over a certain period, notified in case of any increased risk [85], motivated and engaged in using the app, and follow the personalized intervention program. The mHealth app should provide the user with various ways of data visualization and access at any time, scaffolding a participatory management of the disease.

##### Enhanced Security and Privacy Measures

mHealth app developers must ensure that collected user data are secured to maintain the integrity, availability, confidentiality, and resilience of the data [86]. Security procedures should comply with the best practices and regulations (eg, General Data Protection Regulation [79]). Users should be aware of the techniques used to safeguard their personal information and the authentication methods used. These enhanced security measures will make it possible to leverage mHealth tools in daily practice for both clinicians and patients.

##### Improving Participants’ Retention Rates

The success and effectiveness of any mHealth app intervention are directly related to user retention [87]. Therefore, to attain the maximum clinical benefit from the app, designers should ensure that users adhere for the long term to mHealth apps [88]. Various plans could be adopted by designers to re-engage and retain users. mHealth apps should be designed with simple and easy-to-use interfaces as many users refrain from using mHealth apps because of their complicated implementation [89]. Another approach is to continuously notify users about their progress and provide them with positive feedback. An app publisher could also provide users with financial incentives or awards if they achieve a certain healthy goal; for instance, this incentive could be free health insurance or a free subscription to the nearest gym. Applying these design techniques will retain a larger number of participants, resulting in a better impact of the mHealth app interventions.

#### Clinical Recommendations

##### Adoption of Digital Therapeutics

As patients increasingly turn to mHealth apps and devices, clinicians must consider the value of these apps and embrace them to deliver enhanced care. They should adopt more mHealth technologies in their daily practices or workflows and integrate the data into electronic medical records. However, physicians should refrain from recommending apps that have been created without the involvement of medical experts or appropriate testing validation, especially if claims made by app developers are fraudulent. To ensure that their requirements are met and to deliver better outcomes, physicians should actively participate in the design, development, and testing of these mHealth apps [90]. In validated (Food and Drug Administration–approved and *Conformité Européenne*–marked) cases only, *prescription* of these apps can be envisioned, as a form of digital therapeutics, along with wearable devices to allow remote and real-time health monitoring and care delivery [91].

##### mHealth Apps for Communication and Continuous Improvement of Health Care

mHealth apps create a sense of partnership between patients and health care professionals by allowing patients to play a more active role in their health care. Moreover, digital health will improve patients’ engagement with their treatment, something that physicians have previously struggled to do between visits [75]. They also allow proper communication between physicians, patients, and other health care professionals [92]. These tools, assisted by AI and machine learning, represent a rich source of data for clinicians that can be used in medical research to continuously improve the overall delivery of care. It is important to note that these apps are not designed to replace clinicians; on the contrary, they support their decision-making process and workflow. Physicians should not consider these apps as opponents or competitors but rather as an opportunity to enable a streamlined high-quality health care delivery process by capturing and analyzing more data, reaching and monitoring a larger number of patients remotely, and perpetually advancing their clinical practices.

### Limitations

Despite this in-depth analysis, some limitations can be identified in this review. In particular, we refrained from excluding studies based on certain quality criteria, such as study design or sample size, which resulted in large variations in the measurements of outcomes. Moreover, there is a lack of apps that only target osteoporosis; therefore, we included apps that we thought were *useful* for osteoporosis. As no articles in any other language were identified as eligible, the risk of language bias in our selection was negligible. Regarding other biases in the selection, we were cautious in our selection by selecting all related articles in the fields regardless of their outcomes or study design. Finally, some feasibility and development studies were included with the intention of understanding any novel approaches being tested or developed. Such studies seem promising to achieve potential outcomes; however, as these apps were either not tested or tested but with a relatively small sample size, it is difficult to determine whether such solutions can be adopted in the mainstream.

### Conclusions

Given the identified lack of effective mHealth apps with a holistic approach to osteoporosis self-management, this review holds the potential to bridge this gap by proposing a technological tool that goes beyond apps that simply provide information about osteoporosis and creates an individualized care management plan that goes beyond clinical measures. The latter perspective extends the view of mHealth apps from the initial focus on promoting specific behavior, such as healthy nutrition, physical activity, or adherence to medications, to patients’ engagement and empowerment. Moreover, it strengthens collaboration between patients and caregivers by not limiting it to health institutions. In view of the vast quantity of mHealth apps available, it is important for app developers and researchers to identify the proper needs of patients with osteoporosis, adopting a cocreation strategy to create more patient-centered and effective disease management solutions.

## Appendix

Multimedia Appendix 1 PRISMA (Preferred Reporting Items for Systematic Reviews and Meta-Analyses) checklist, detailed search terms, and sensitivity analysis.

## Abbreviations

AI: artificial intelligence
BMD: bone mineral density
mHealth: mobile health
PRISMA: Preferred Reporting Items for Systematic Reviews and Meta-Analyses
RCT: randomized controlled trial
ROB: risk of bias
